# Rare Variant Analysis for Family-Based Design

**DOI:** 10.1371/journal.pone.0048495

**Published:** 2013-01-15

**Authors:** Gourab De, Wai-Ki Yip, Iuliana Ionita-Laza, Nan Laird

**Affiliations:** 1 Department of Biostatistics, Harvard University, Boston, Massachusetts, United States of America; 2 Department of Biostatistics, Columbia University, New York, New York, United States of America; University of Texas School of Public Health, United States of America

## Abstract

Genome-wide association studies have been able to identify disease associations with many common variants; however most of the estimated genetic contribution explained by these variants appears to be very modest. Rare variants are thought to have larger effect sizes compared to common SNPs but effects of rare variants cannot be tested in the GWAS setting. Here we propose a novel method to test for association of rare variants obtained by sequencing in family-based samples by collapsing the standard family-based association test (FBAT) statistic over a region of interest. We also propose a suitable weighting scheme so that low frequency SNPs that may be enriched in functional variants can be upweighted compared to common variants. Using simulations we show that the family-based methods perform at par with the population-based methods under no population stratification. By construction, family-based tests are completely robust to population stratification; we show that our proposed methods remain valid even when population stratification is present.

## Introduction

It is now widely accepted that many diseases are caused by a complex interplay between multiple genes and other non-genetic factors, and that different genetic susceptibility factors may be responsible for disease risks in different individuals. Genome-wide association studies have been able to identify many variants associated with complex diseases that are common in the population. However most of the estimated genetic contribution explained by these common variants appears to be very modest. On the other hand, rare variants are thought to have a larger effect size compared to common SNPs [1]. Availability of sequencing data from specific candidate genes and functional genomic regions such as exons for a large number of individuals and from whole genome for a smaller set of individuals [2], has made it possible to gain a wealth of information about the potential effect of multiple rare variants on complex phenotypes. Conventional statistical methods for common variants have low power for low frequency SNPs, particularly when the power relies on the linkage disequilibrium (LD) between the causal variants and the observed markers.

To overcome the problem of poor power of the single SNP strategy, the strategy of collapsing rare variants over a gene and collective analyses of their association has been adopted. Morgenthaler et al [3] devised the CAST (cohort alleleic sum test) which collapses over the rare variants and then compares the total rare variant frequency between cases and controls. Li and Leal [4] extended the CAST method to come up with CMC (combined multivariate and collapsing) method where collapsing is done within different subgroups defined by allele frequencies and combined using a multivariate distance-based statistic. All these methods use a fixed threshold for specifying rare variants i.e. the user must define a value for allele frequency to distinguish between rare and common variants.

Madsen and Browning [5] proposed a method whereby variants of any frequency can be included, but the variants are weighted according to their frequencies - thus allowing rare variants to contribute more to the test statistic than they do in the unweighted case. Price et al [6] proposed a variable threshold approach, designed to eliminate the need of choosing a fixed threshold to include variants and showed that this method can be more powerful compared to the fixed threshold approach. Moreover they also proposed ways to include information about functional impact of the variants. Hoffmann et al [7] introduces weights which can incorporate allele frequency, direction (deleterious or protective) and threshold all in a single analysis. More recently Lin and Tang [8] proposed a general framework based on appropriate regression methods to cover a wide range of study designs.

More recent methods deal with some of the more complex issues of pooled rare variant analysis. Zhu et al [9] uses a haplotype-based method to identify relevant rare haplotypes associated with the disease. Hoffmann et al [7] uses a ‘step-up’ approach similar to forward selection to identify an optimal grouping of rare variants. Ionita-Laza et al [10] suggests a replication-based weighted-sum statistic which is applied separately to potential risk variants (those with observed higher frequency in cases compared with controls) and potential protective variants, and Neale et al [11] tailored the C-alpha [12] score test to test for rare variants association - both of the two methods can address the case where variants have different direction of effects on the same genetic region. Lin and Tang [8] introduce a general score-based test for population-based samples, that unifies many of these approaches.

Even though there is a considerable sum of literature on methods for rare variant analysis, few discuss family-based designs. Family-based analysis for single SNP association uses information about transmission of genetic factors within families and has been shown to potentially have more power than the population-based design for rare diseases [13]. Moreover the family-based design is robust to any bias induced by population substructure. For rare variants this issue of population stratification is even more acute as rare variants may include young mutations that are more population-specific [2], [14]. In this paper we propose a novel method to test for association of rare variants in family-based design by extending the traditional single SNP Family-Based Association Test. To include both common and rare variants in our analysis, we introduce suitable weighting schemes to upweight rarer variants and downweight the more common variants. We also evaluate the analysis using a fixed threshold value to identify the rare variants. Finally we compare performance of these methods under different settings against the fixed threshold version of the population-based score statistic by Lin and Tang [8] and the population-based weighted sum statistic by Madsen and Browning [5].

Our approach builds on the FBAT multimarker test [15], which is a ‘gene-based test’ designed for testing multiple SNPs from a GWAS or candidate gene strictly. The proposed test is also similar to FBAT-LC statistic, [16] which was designed for powerful and efficient multimarker testing for measured phenotypes. Relationship among these statistics are discussed in the subsequent section.

## Methods

### Family-based Association Test

We first consider a sample of 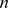 trios - one offspring with information on both parents available and review the single variant setting. The general FBAT statistic is a covariance between the offspring genotype and trait. Let 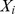 and 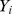 denote the genotype for the variant and the trait, respectively, for the 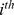 offspring. In the general case, 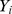 can be both measured or dichotomous, and we can use an offset 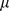 to appropriately center the trait [17]. For family samples with dichotomous traits such as affected trios or discordant sibpairs, 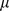 is often taken to be zero; with measured outcomes, mean of the outcome is usually chosen for offset. For the additive model, 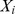 is the number of copies of minor alleles for the locus of interest. We define
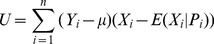
(1)

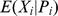
 in (1) is computed using Mendel’s laws under the null hypothesis of no association and conditional on the trait as well as the parental genotypes (denoted as 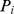 for the i-th family). Under the same conditional distribution, we can compute Var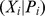; the large sample FBAT statistic is defined as
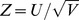
(2)where 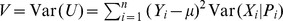. Under the null hypothesis of no association Z is approximately N(0,1). The formula extends easily where multiple offspring are sampled in a family for testing the null hypothesis of no association and no linkage.

The FBAT Multi-Marker test is a multivariate extension of the univariate FBAT test designed to simultaneously test a set of markers in a defined region, such as a gene. It belongs to the general class of ‘gene-based tests’ since a set of M univariate tests in a gene are replaced by a single multivariate test. Let 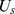 and 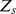 denote the statistics in equation 1 and 2, defined for the 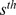 marker. Assuming large samples to obtain sufficient heterozygote parents, each 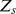 is approximately N(0,1), but the M markers may be correlated because of linkage disequilibrium in the region. Provided we have an estimate of the correlation matrix, we can obtain a M degree of freedom test of the null hypothesis of no association between any of the M variants and the disease, versus the alternative that at least one marker is in LD with a disease locus.

Rakovski et al [15] estimate the correlation matrix empirically as follows: Let 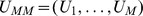 be the vector of FBAT statistics, which forms the basis of the multimarker test. Let 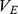, the empirical variance estimator, be the 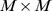 matrix with elements
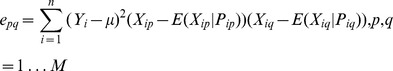
and 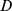 be the diagonal matrix with elements equal to the Var(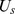)’s where 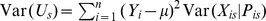. The corresponding adjusted variance matrix 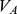 is defined by



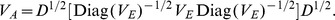



Note that 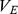 is a variance-covariance matrix, with all elements estimated empirically. However the diagonal elements of 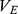 can be calculated directly provided there is no linkage between any marker and the true disease locus. 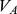 is an ‘adjusted’ variance covariance matrix which replaces the empirical variances with the exact ones. The multi-marker test is then defined as
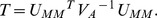



In large samples, T will be approximately 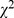 distributed with degrees of freedom equal to the rank of 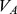. The asymptotic normality relies on the asymptotic normality of each marker test 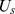, and may not be valid in the rare variant setting.

Several papers have noted that tests of multiple markers can be greatly improved upon by taking optimal linear combinations of the individual tests [8], [16], [18], [19], but a major issue is determining the optimal weights, since the optimal weights depend upon the unknown effect of each marker. Xu et al [16] proposed a method to handle this problem by using that portion of the family data that is not used in constructing the FBAT statistics, e.g. the noninformative families [13],[20]. The approach is designed for measured outcomes, or at least cases where both affected and unaffected offspring are sampled. The approach can be extended in principle to the setting where we have only affected trios [21], but this is beyond the scope of this paper. An additional feature of the FBAT-LC approach is that estimation of the weights can be invalidated by population substructure.

### Collapsing Method for Rare Variants

We extend the general FBAT statistic to test for rare variants by using the approach of collapsing over a gene or a particular genetic region. We assume that any variants associated with the trait have effects in the same direction. Let 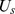 be the FBAT statistic corresponding to the 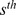 variant and M be the total number of variants in the region. We define
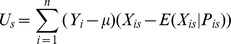
where 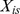 is the number of copies of 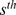 variant in 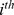 offspring. Then the unweighted FBAT statistic for rare variants can be defined as



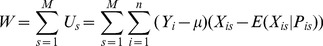
(3)If we change the order of the summations we can express the test statistic as
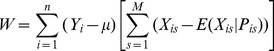
which is essentially a covariance between the trait and total number of copies of all variants in a region. The variance of W can be expressed as



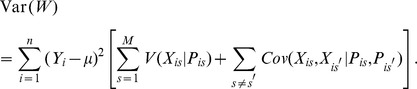



The 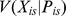
’s are the variances of each of the M SNPs conditional on the trait and the parental genotypes; they can be computed under the null hypothesis of no association simply using Mendel’s laws as applied to the transmission probabilities.

As previously discussed, 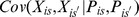 can be estimated empirically using the variance estimator for multimarker FBAT statistic. One advantage of using this method is that it avoids haplotype reconstruction. As W can be expressed as 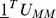, a suitable estimate for Var(W) is 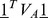 = the sum of all elements in 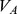 (as computed for the multimarker statistic). The standardized test statistic
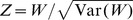
is approximately N(0,1) in large samples under the null hypothesis of no association. Note that asymptotic normality here only relies on the normality of W, which should hold in the rare variant setting provided the number of variants in the tested region is not small.

Extension of the test statistic for other nuclear family structures is straightforward. For a large multi-generation pedigree, the simplest strategy would be to break the pedigree into nuclear families and combine the contribution from all those nuclear families. For example, the general FBAT statistic for the 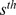 variant for a family with multiple offspring can be defined as
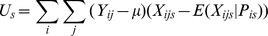
where the summand corresponds to the 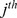 offspring of the 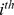 family. We can use this statistic to get the collapsed statistic 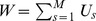. When partial or no information is available on the parental genotype, 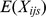 and Var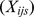 are computed conditional on the trait values as well as the sufficient statistics for parental genotypes. Note that the computation of variance in such cases follows directly from computation of the variance estimator for multimarker FBAT statistic. In this paper we will consider the analysis for two common family designs - trios and discordant sibpairs (DSP).

### Weighting Scheme

When the region of interest contains both common and rare variants, improvement in the performance of the statistic in detecting effects of very low frequency variants will require choosing a suitable weighting scheme that would upweight the variants with lower frequencies. If the 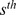 variant is assigned the weight 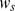, the weighted FBAT statistic for rare variants can be defined as
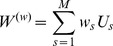
(4)


For our purpose we use two sets of weights : 1) 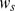 = 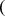
# of informative families for 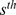 variant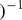, where informative families for trios are defined as families with at least one of the parents being heterozygous at the locus, and 2) 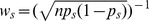 where n is the total number of nuclear families and 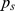 is the allele frequency for 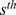 variant estimated from the sample. The latter weighting method is similar to the weighting scheme used by Madsen and Browning [5] where they estimate the allele frequencies from the control population. As argued in context of their method, the weight of a variant is inversely proportional to the estimated standard deviation of the number of alleles of the corresponding marker in a random sample selected from the population under the null distribution of no association. In this case, for trios, allele frequencies are estimated from parents, and for discordant sibpairs, allele frequencies are estimated from all siblings. The weights in both cases are independent of the transmissions used in the test statistic since they depend only on parental genotypes, or the sufficient statistics for parental genotypes in the case of discordant sibpairs [22]. Implicitly these weights assume all markers have effects of the same sign, and that the magnitude of the effect increases as corresponding allele frequency decreases.

Weighting schemes other than these two options can also be used if external information is available about the SNPs. Price et al [6] suggested using functional predictions about the SNPs using Polyphen-2 [23] scores to weight the SNPs. Similarly SIFT [24] or other functional prediction scores can also be incorporated to compute weights.

As discussed previously, Xu et al [16] also proposed a weighted linear combination of FBAT statistics (FBAT-LC) in the context of finding an optimal test for associating a set of SNPs with a quantitative trait. This is similar to what Lin and Tang [8] propose for estimating the effect of each SNP, except the weights are estimated independently of the test statistics by regressing the trait on the expected marker score using the conditional mean model [13]. FBAT-LC is not available for trios with affected only, but would make an attractive extension for the setting where the effects of disease SNPs vary sign as well as magnitude.

### Fixed Threshold Approach

When the region of consideration contains both rare and common variants, instead of weighting the variants differently based on their allele frequencies, we can also use a user-defined threshold value based on allele frequencies to identify a subset of variants as rare and consider analysing only that subset. The fixed threshold collapsed sum FBAT statistic is defined as
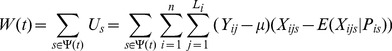
(5)where 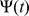 is the subset of SNPs that have allele frequencies less than the threshold value t. Conventional values of t, used to define rare variants, are 1% and 5%. In this case, we assume that only rare variants are associated with the trait - however the analysis depends on a suitable choice of threshold value.

### Simulation

The simulations are based on the assumption that variants in the region of interest are under weak purifying selection and Wright’s distribution is used to sample frequency for each variant
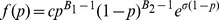
where 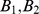 are scaled mutation rates and 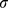 is the selection rate. We take 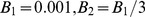 and 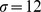 as in Madsen and Browning [5]. We fix the number of variants in the region to be 50 and we randomly select the DSVs out of the variants that have frequency less than 1%. We choose the number of DSVs to be 10 or 20.

We generate the binary trait using a relative risk model for the DSVs. We assume
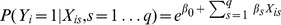
(6)where 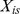 is the number of copies of 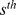 variant in 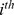 individual and q is the number of DSVs. We examine two set-ups - in the first set-up all variants have equal 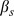
’s. In the second set-up we vary the effect of the DSVs within a range of values exponentially such that a lower frequency DSV is associated with a higher effect - we use the formula 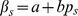 for a value of a and b (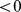), where 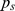 is the s-th variant frequency.

We generate samples from the trio design as well as sibpairs. We generate parental haplotypes using the frequencies for the variants assuming the variants to be independent. Once the parental haplotypes are generated offspring haplotypes are generated from the parental haplotype assuming no recombination between the variants. Once offspring haplotypes are generated we only use the genotypic data for simulating the disease status as well as for the analysis. For the trio design we use offset 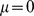 i.e. only affected offspring are used in our analysis and the total number of affected offspring is fixed (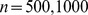). Similarly for sibpairs we only use discordant sibpairs in the analysis, fixing the total number of discordant sibpairs (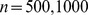). For the case-control design, we use the affected offspring (generated for the trio design) as cases and select an equal number of controls from the unaffected offspring (from different families).

For analysis we use both the fixed threshold approach and the no threshold approach. In this paper we use 0.5%, 1% and 5% as cutoff values for fixed threshold analysis where the true DSVs have frequencies less than 1%. Performance of the FBAT-multimarker (FBAT-MM) is also evaluated as a fixed threshold test for these threshold values. Note that since FBAT-MM is a multivariate test, it’s not straightforward to construct a weighted version of the test statistic. For the no threshold approach, we analyze all variants in the region of interest. We evaluate performance of our method under the trio design as well as the sibpair design and compare the method against the fixed threshold method by Lin and Tang [8] and a modified version of the weighted-sum statistic by Madsen and Browning [5] for the case-control design. For both the case-control methods, frequency of the variants is estimated using both cases and controls based on recommendation by Lin and Tang [8] - it should be noted that this method of estimating frequency is different from traditional way used in Madsen and Browning [5] which uses only controls to estimate allele frequency. We compare both type-1 error as well as power under different settings.

Moreover we also investigate the effect of population stratification which is potentially a problem for rare variants analysis. To introduce stratification, first we simulate two subpopulations with different distribution for the risk variants - for the first subpopulation DSV allele frequencies are generated from the Wright’s distribution above and for the second population corresponding frequencies for DSVs are generated using the Balding-Nichols model, [25] ensuring that the expected value of the fixation index (FST) of the population is 0.01 or 0.05 - only the results related to FST = 0.01 is shown here as FST = 0.05 is a more extreme case of population stratification. Next, different values for the baseline risk or 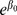 = 0.05 or 0.01 are used for the two subpopulations.

## Results

Among the two weighting schemes, weighting by allele frequency estimates performs substantially better than weighting by number of informative families, so we only display results related to the former. Table 1 contains descriptive statistics for estimates of prevalence (P(Y = 1)) and total population attributable rate (PAR) for all 50 variants, computed from 500 simulations using 500 cases and 500 controls. Note that we use the affected offspring (generated for the trio design) as cases and select an equal number of controls from the unaffected offspring (from different families). Total PAR for the genetic region is defined as the sum of PARs over all DSVs, where
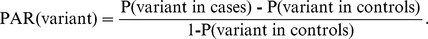



**Table 1:** Mean and standard deviation of estimates of prevalence and population attributable fraction(PAR) from 500 simulations for different values of the true relative risk (RR) associated with a variant, # of cases = 500 and no population stratification.

DSV	RR( 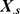 )( = 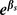 )	Prevalence [Mean (SD)]	PAR [Mean(SD)]
10	1	0.050(0.002)	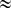 0(0.005)
	2	0.051(0.002)	0.012(0.010)
	3	0.052 (0.003)	0.024 (0.016)
20	1	0.050(0.002)	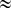 0(0.007)
	2	0.052(0.003)	0.026(0.015)
	3	0.055(0.003)	0.050(0.023)

It can be seen that the overall prevalence is very close to 0.05, the baseline risk value used in the simulation and the estimated average PAR is between the range 0–0.05.

Table 2 compares the type-1 error for the family-based methods for fixed threshold (unweighted) and no threshold (weighted by allele frequencies) with the corresponding case-control based methods (Lin and Tang [8] method and the modified version of Madsen-Browning [5] respectively) and the FBAT-MM test. At the 5% and 1% level with no population stratification all the tests controlled the type-1 error well. The Q-Q plots (not shown here) suggested that type-1 error was controlled at lower levels as well. However when population stratification was introduced, all the population-based statistic had a significantly high false positive rate. For family-based tests, the p-values were well-controlled. The type-1 errors were also well controlled under the sibpair design (not shown here). FBAT-MM is very conservative; at alpha of 0.05, the degree of conservativeness increases as the threshold lowers.

**Table 2:** Type-1 error using normal cut-off at 0.05 and 0.01 level for 500 trios.

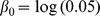 No population stratification,														
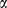	Fixed threshold Family-based*					Fixed threshold FBAT-MM*				Fixed threshold Case-control*			No threshold Family-based	No threshold Case-control
	0.005		0.01		0.05	0.005	0.01		0.05	0.005	0.01	0.05		
0.05	0.050		0.046		0.046	0.014	0.020		0.030	0.048	0.052	0.054	0.050	0.028
0.01	0.006		0.010		0.010	0.002	0.002		0.002	0.008	0.004	0.010	0.006	0.008
**Population stratification, 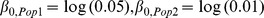 , FST = 0.01**														
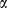		**Fixed threshold Family-based** *				**Fixed threshold FBAT-MM** *				**Fixed threshold Case-control** *			**No threshold Family-based**	**No threshold Case-control**
		0.005	0.01	0.05		0.005		0.01	0.05	0.005	0.01	0.05		
0.05		0.048	0.042	0.048		0.018		0.037	0.033	0.260	0.220	0.106	0.048	0.092
0.01		0.014	0.008	0.020		0		0.002	0	0.100	0.086	0.036	0.008	0.034

* Threshold values 0.005, 0.01 and 0.05 were used for the fixed threshold methods.

**Figure 1:**
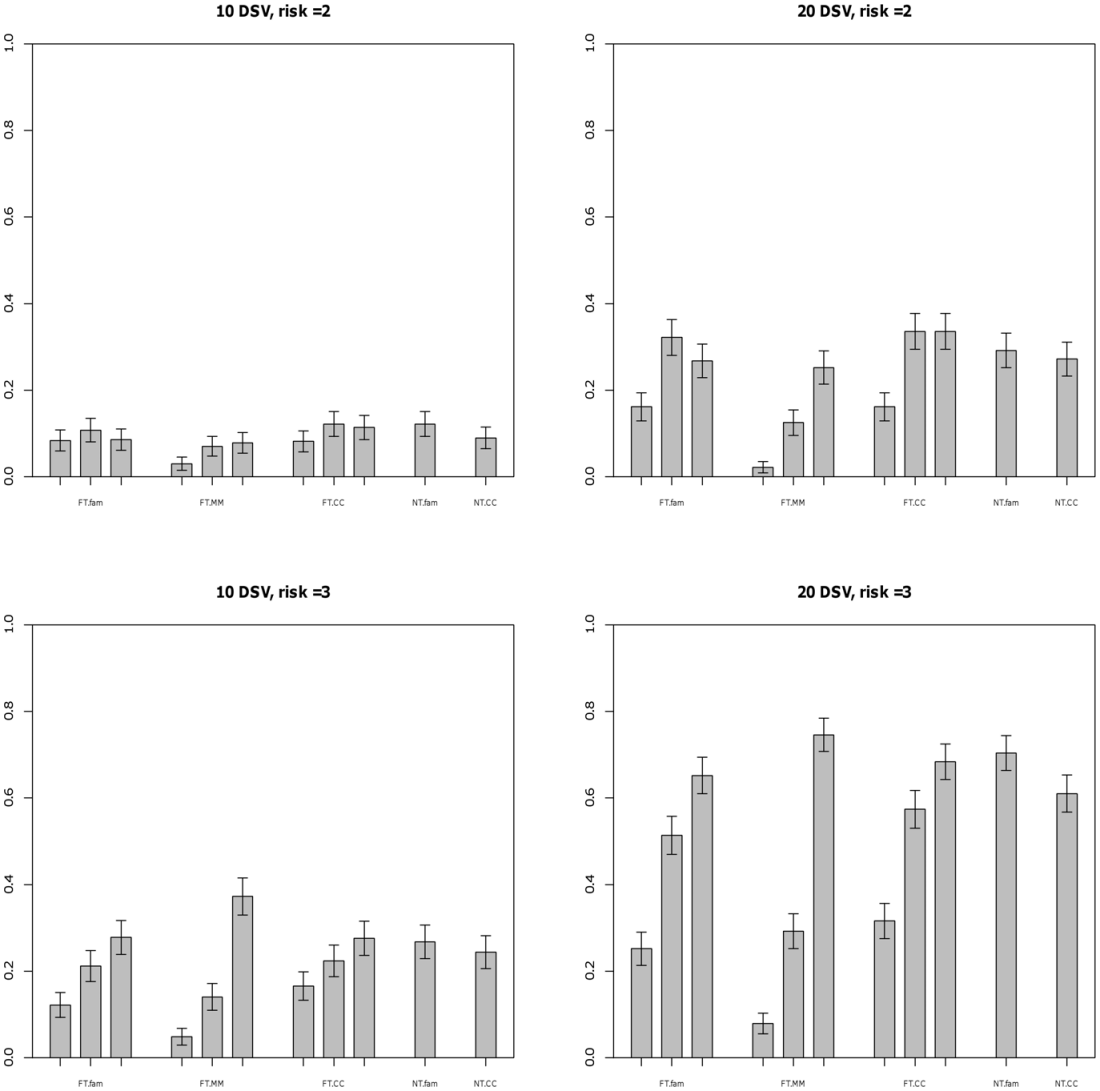
Power at 0.05 level for trios and case-control design - No population stratification is present and 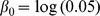. # of cases = 500. DSV’s have frequency less than 0.01 and equal effects. FT.fam - trios with fixed threshold method using threshold 0.005, 0.01 and 0.05, FT.MM - FBAT-MM test using threshold 0.005, 0.01 and 0.05, FT.CC - case-control with unweighted method using threshold 0.005, 0.01 and 0.05, NT.fam - trios with weighted method using no threshold, NT.CC - case-control with Madsen and Browning method.

Figure 1 displays relative performance of the methods when no population stratification is present and effects of all the DSVs are equal. The number of affected subjects in these simulations is 500. For the fixed threshold method, using a lower threshold value (0.5%) in contrast to using the true threshold for DSV (1%) incurred lower power in all settings for the unweighted method. Analysis using a higher threshold value (5%) performed at par with and in some setting better than analysis using the true threshold value (1%). FBAT-MM method performed slightly better compared to the proposed family-based methods when magnitude of the effect was higher. Performance of the proposed family-based methods were similar to the case-control method for the fixed threshold approach and better than the case-control method when weighted methods were used. Under admixture, the power of the case-control methods increase due to the anti-conservativeness, but the power of the family-based methods is unchanged (Figure S1).

**Figure 2:**
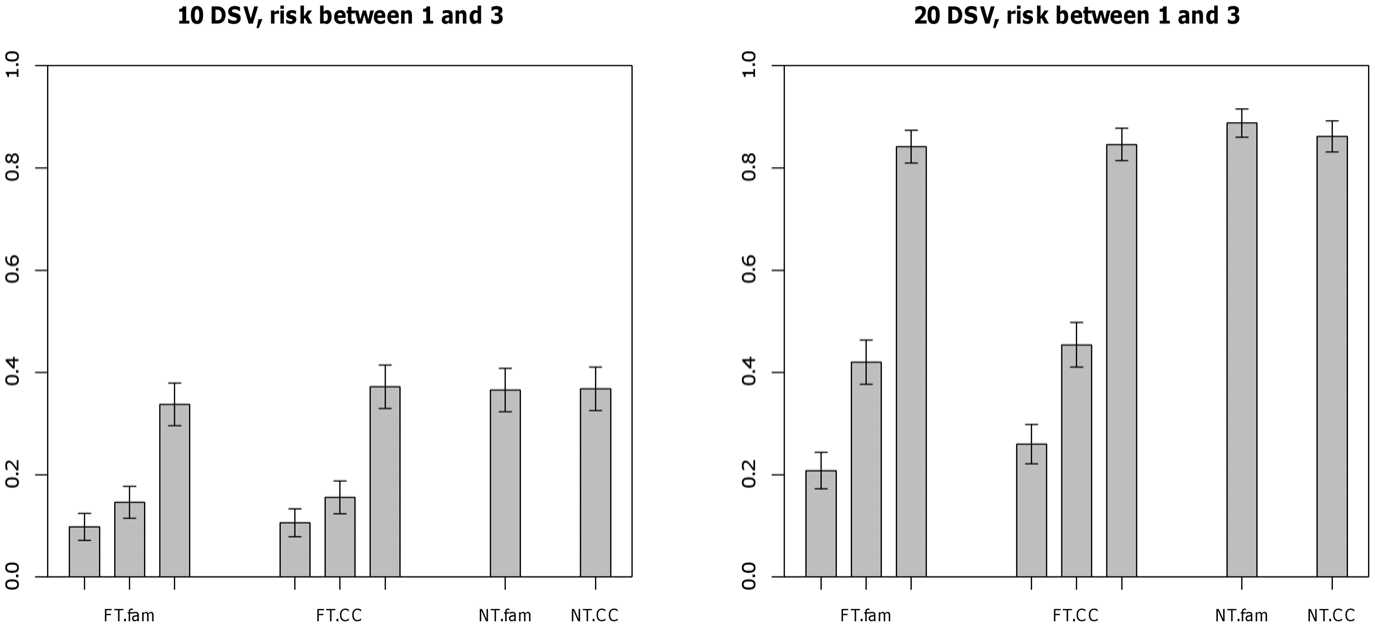
Power at 0.05 level for trios and case-control design - No population stratification is present and 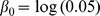. # of cases = 500. DSVs have frequency less than 0.01 and varying effects. FT.fam - trios with fixed threshold method using threshold 0.005, 0.01 and 0.05, FT.CC - case-control with unweighted method using threshold 0.005, 0.01 and 0.05, NT.fam - trios with weighted method using no threshold, NT.CC - case-control with Madsen and Browning method.

**Figure 3:**
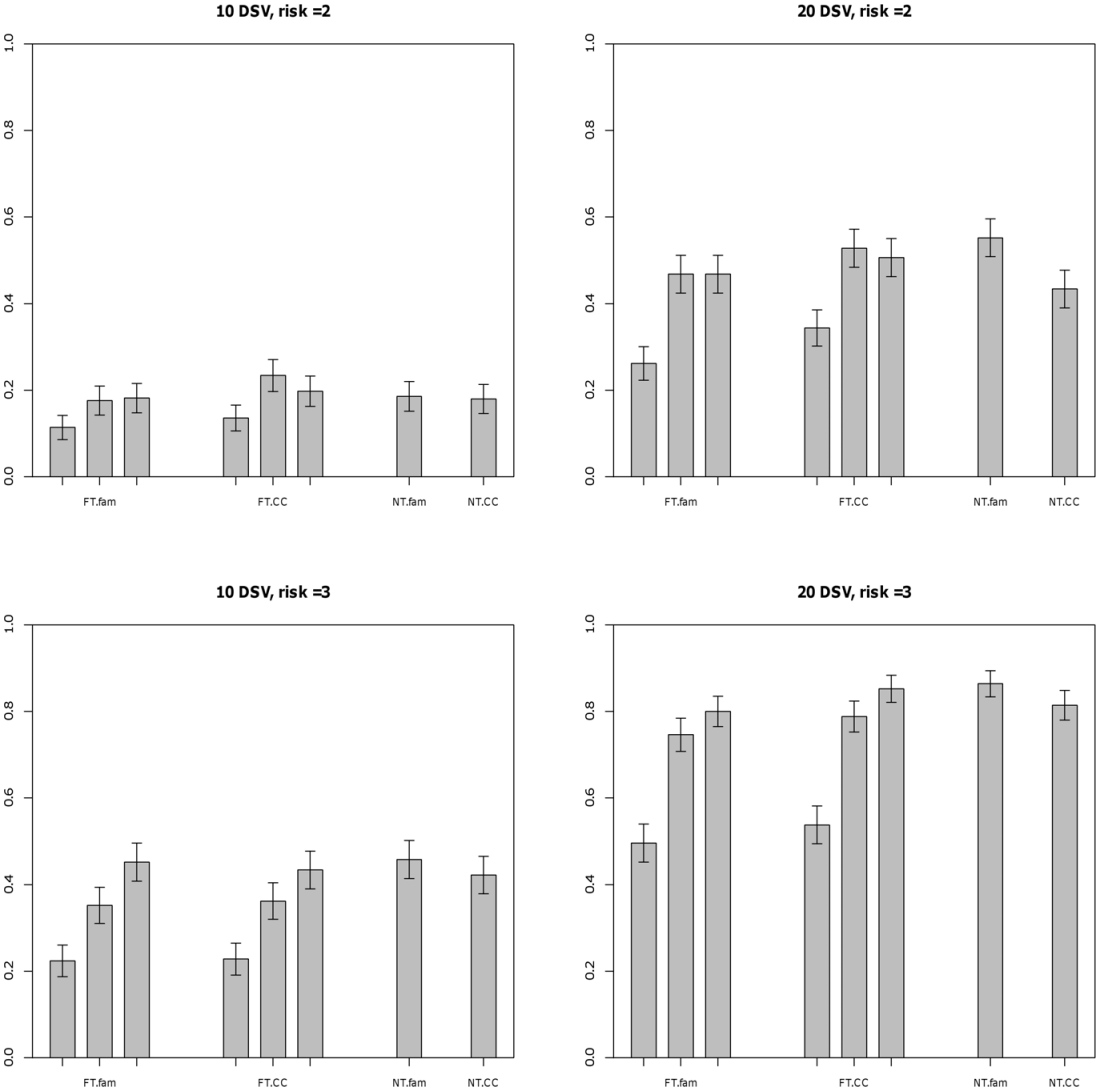
Power at 0.05 level for trios and case-control design - No population stratification is present and 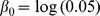. # of cases = 1000. DSV’s have frequency less than 0.01 and equal effects. FT.fam - trios with fixed threshold method using threshold 0.005, 0.01 and 0.05, FT.CC - case-control with unweighted method using threshold 0.005, 0.01 and 0.05, NT.fam - trios with weighted method using no threshold, NT.CC - case-control with Madsen and Browning method.

Figure 2 shows the power in the case where the effect size of an allele is inversely related to it’s frequency. The results are generally unchanged, and the higher power of the 5% threshold is clearly present for both family and case-control designs. Figure 3 shows the results for 1000 trios and 1000 cases. The power is higher as expected, and the general trends seen for 500 trios or cases are the same, except that the difference between the 1% and 5% thresholds are now much smaller. The 0.5% threshold continues to have low power. This suggests that the higher power for the 5% threshold is due to sampling error in the estimation of allele frequency; the threshold is compared to the estimated sample frequency. Under 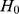, the probability that an allele frequency of 1% would be estimated as greater than 1% with 500 trios is quite low, but will increase under selection of cases only when the alternative hypothesis is true, especially for higher effect sizes. In general, the power of the threshold approaches depend strongly on the selected threshold, the no threshold weighted methods were at a par with the best of the threshold approaches, and the family and case-control designs showed little difference in power. The results for the sib-ship design (Figures S4, S5) show that it has considerably lower power than the trio design, as we might expect from previous comparisons in the case of common DSVs [13].

## Discussion

In this paper we present a family-based method designed specifically for rare variants. Family-based design are robust from bias due to population substructure and is particularly useful for rare variant analysis since the issue of population stratification is more prominent for rare variants. Moreover since genotyping error rates are still substantially high for sequencing technologies, family-based design provides the additional advantage of checking for Mendelian error [26],[27]. Power simulations show that the proposed methods perform reasonably well compared to existing population-based methods. Both the weighted and the unweighted methods for family-based design preserves the type 1 error when population stratification is present.

Analysis of rare variants can be approached as a separate problem from analysis of common variants i.e. after analysis of all common variants in a genetic region, only rare variants can be analyzed to look for an association signal. In this paper the performance of unweighted method using a fixed threshold approach illustrates the situation where a select group of rare variants are analyzed. However the performance of the fixed threshold approach is highly dependent on the choice of the threshold value and such dependence remains even when a weighting scheme is applied along with the fixed threshold (not shown here). Choice of a threshold value which is lower than the true threshold value (i.e. the cutoff for the true DSVs) incurs substantially low power in almost all settings. The choice of the optimal threshold depends upon the unknown allele frequency and effect size of the DSLs, as well as the sample. The weighted no threshold approach avoids such choice problem. Moreover when a suitable weighting scheme is used, the no threshold approach performs at par with and sometimes better than the best performing fixed threshold approach, especially when the variants have unequal effects. This approach always outperformed the corresponding no threshold case-control method i.e. the modified Madsen-Browning approach. Hence we recommend using a weighted approach with no threshold in the absence of any prior knowledge about the frequency of the DSVs.

In this paper we have used a weighting scheme based on allele frequency but other weighting schemes based on functional information can also be used. It should be noted that, even though in this paper we suggest a few different weighting schemes, the optimal weighting scheme is unknown and dependent on the underlying disease model. The user can possibly use an omnibus test, where p-values are minimized over different choices of weighting schemes. Another alternative to the fixed threshold method can be using the variable threshold method suggested by Price et al [6] but this would involve a permutation-based test which can be complicated for ascertained family-based samples.

As noted earlier, our method uses similar components as the FBAT multimarker test but there are a few key differences. FBAT-MM was designed to analyze GWAS data or a fixed region such as candidate genes. Since the degree of freedom in FBAT-MM test is the number of markers being analyzed, power of this test may be adversely affected when applied on sequence data with high number of variants. In addition, the multimarker test statistic requires a stricter assumption of normality compared to our proposed method and seemed to be quite conservative in terms of type-1 error under low threshold values. Despite these caveats, FBAT-MM performed very well in the fixed threshold setting. An attractive extension would be to develop a weighted method for the no threshold approach. This should work especially well in the setting where the DSL’s have both positive and negative effects.

A major issue associated with any collapsing based method is the issue of variants in a region having different direction of effect on the trait. This paper focuses only on the situation where all the variants have effect in the same direction. However, if there are variants with opposite direction (a mix of deleterious and protective variants) the collapsing method can lead to lower power. It should be noted that this particular case might have less impact on the power of FBAT-MM. One way of improving upon the proposed family-based statistic could be to construct two different statistic for the two different direction of variants based on the sign of their estimated effects, following the strategy used by Ionita-Laza et al [10]. This issue has not been addressed within the scope of this paper however it warrants further investigation.

Most rare variant association analyses are largely motivated by the idea that variants under 1–5% allele frequency have a higher proportion of functional variants [28]. Hence we assume that the disease suseptibility variants have allele frequency less than 1%. This is, however, an extreme case of alleleic heterogeneity and in more realistic situation common variants can also be DSV’s.

Performance of the method has been illustrated in this paper for binary traits only - however we can easily extend the method for quantitative traits. For this setting permutation testing strategies are also available which is beyond the scope of this paper. Moreover in this paper, we have not discussed any existing methods for correcting bias due to population stratification for population-based methods. Most of these methods (e.g. principal component correction) have been originally developed for genome-wide association studies and could be extended to sequence data in principle. But feasibility of these methods for small genetic region has not been studied in detail. Moreover a recent study [29] suggests that for rare variants these existing method show suboptimal performance in correcting for population stratification.

In this paper we only analyze nuclear families and we estimate variance of the test statistic using empirical estimates for covariance between markers summing the contributions from the nuclear families. This estimation method can be inefficient when the method is being used for a multi-generation pedigree [30] or distant set of relatives. Secondly, we have used an additive model for SNPs for the analysis - other models (dominant or recessive) need to be investigated as well. Since for rare variants dominant and additive models tend to be very similar, we suspect the trends to be similar as well. For recessive models, we suspect the power of all the methods to be smaller, except when the true disease model is recessive. Thirdly, our results show that the trio design is more powerful than a balanced case-control design with equal number of cases but the discordant sibpair design is far less powerful. This will incur a higher genotyping cost for nuclear families compared to population-based methods to achieve comparable power.

Both the unweighted and weighted method for rare variants are included as options in the FBAT package. It is currently available as beta release and will be officially released soon in the future.

## Supporting Information

Figure S1
**Power at 0.05 level for trios and case-control design - Mixture of two subpopulations, 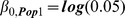, 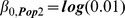, FST = 0.01.** # of cases = 500. DSV’s have frequency less than 0.01 and equal effects. FT.fam - trios with fixed threshold method using threshold 0.005, 0.01 and 0.05, FT.CC - case-control with unweighted method using threshold 0.005, 0.01 and 0.05, NT.fam - trios with weighted method using no threshold, NT.CC - case-control with Madsen and Browning method. It should be noted that the inflated power for case-control methods are also associated with inflated type-1 error under population stratification.(DOC)

Figure S2
**Power at 0.05 level for trios and case-control design - Mixture of two subpopulations, 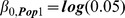, 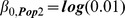, FST = 0.01.** # of cases = 500. DSVs have frequency less than 0.01 and varying effects. FT.fam - trios with fixed threshold method using threshold 0.005, 0.01 and 0.05, FT.CC - case-control with unweighted method using threshold 0.005, 0.01 and 0.05, NT.fam - trios with weighted method using no threshold, NT.CC - case-control with Madsen and Browning method. It should be noted that the inflated power for case-control methods are also associated with inflated type-1 error under population stratification.(DOC)

Figure S3
**Power at 0.05 level for trios and case-control design - Mixture of two subpopulations, 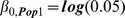, 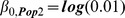, FST = 0.01.** # of cases = 1000. DSV’s have frequency less than 0.01 and equal effects. FT.fam - trios with fixed threshold method using threshold 0.005, 0.01 and 0.05, FT.CC - case-control with unweighted method using threshold 0.005, 0.01 and 0.05, NT.fam - trios with weighted method using no threshold, NT.CC - case-control with Madsen and Browning method. It should be noted that the inflated power for case-control methods are also associated with inflated type-1 error under population stratification.(DOC)

Figure S4
**Power at 0.05 level for discordant sibpairs - No population stratification is present and 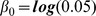.** # of cases = 500. DSV’s have frequency less than 0.01 and equal effects. FT.fam - sibs with fixed threshold method using threshold 0.005, 0.01 and 0.05, NT.fam - sibs with weighted method using no threshold.(DOC)

Figure S5
**Power at 0.05 level for discordant sibpairs - Mixture of two subpopulations, 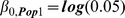, 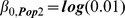, FST = 0.01.** # of cases = 500. DSV’s have frequency less than 0.01 and equal effects. FT.fam - sibs with fixed threshold method using threshold 0.005, 0.01 and 0.05, NT.fam - sibs with weighted method using no threshold.(DOC)
