# Supplementary material for: Rare Variant Analysis for Family-Based Design
Source: PLoS One. 2013 Jan 15;8(1):e48495. doi: 10.1371/journal.pone.0048495 (PMC3546113; doi:10.1371/journal.pone.0048495)

**Figure S2.** Power at 0.05 level for trios and case-control design - Mixture of two subpopulations, β_0,Pop 1_ = log(0.05), β_0,Pop 2_ = log(0.01), FST = 0.01. # of cases= 500. DSVs have frequency less than 0.01 and varying effects. FT.fam - trios with fixed threshold method using threshold 0.005,0.01 and 0.05, FT.CC - case-control with unweighted method using threshold 0.005,0.01 and 0.05, NT.fam - trios with weighted method using no threshold, NT.CC - case-control with Madsen and Browning method. It should be noted that the inflated power for case-control methods are also associated with inflated type-1 error under population stratification.


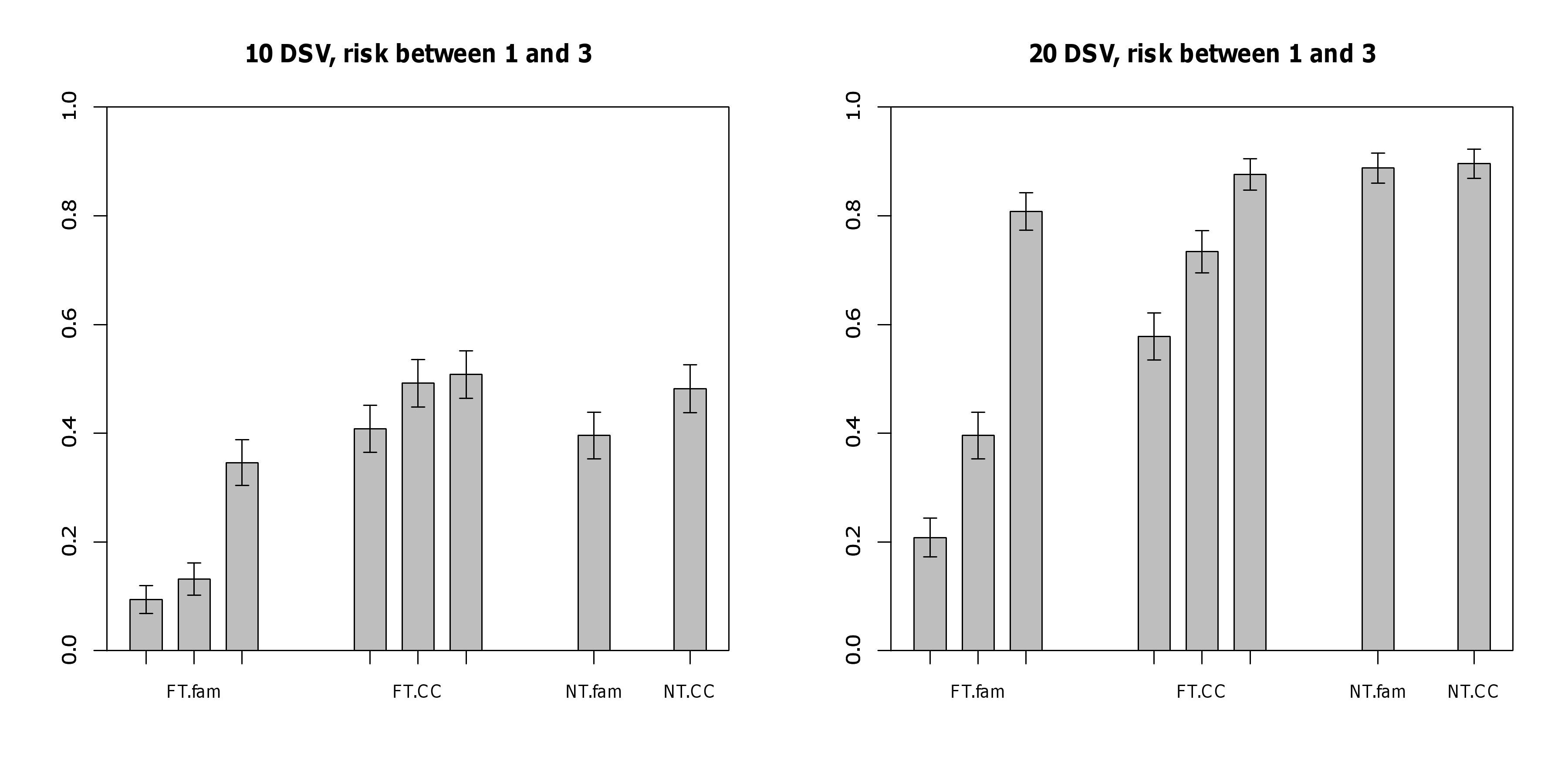

Supplement: Figure S2 — Power at 0.05 level for trios and case-control design - Mixture of two subpopulations, , , FST = 0.01. # of cases = 500. DSVs have frequency less than 0.01 and varying effects. FT.fam - trios with fixed threshold method using threshold 0.005, 0.01 and 0.05, FT.CC - case-control with unweighted method using threshold 0.005, 0.01 and 0.05, NT.fam - trios with weighted method using no threshold, NT.CC - case-control with Madsen and Browning method. It should be noted that the inflated power for case-control methods are also associated with inflated type-1 error under population stratification. (DOC) [file pone.0048495.s002.doc]
