# Supplementary material for: Rare Variant Analysis for Family-Based Design
Source: PLoS One. 2013 Jan 15;8(1):e48495. doi: 10.1371/journal.pone.0048495 (PMC3546113; doi:10.1371/journal.pone.0048495)

**Figure S4.** Power at 0.05 level for discordant sibpairs - No population stratification is present and β_0,Pop 1_ = log(0.05). # of cases= 500. DSV's have frequency less than 0.01 and equal effects. FT.fam - sibs with fixed threshold method using threshold 0.005, 0.01 and 0.05, NT.fam - sibs with weighted method using no threshold.


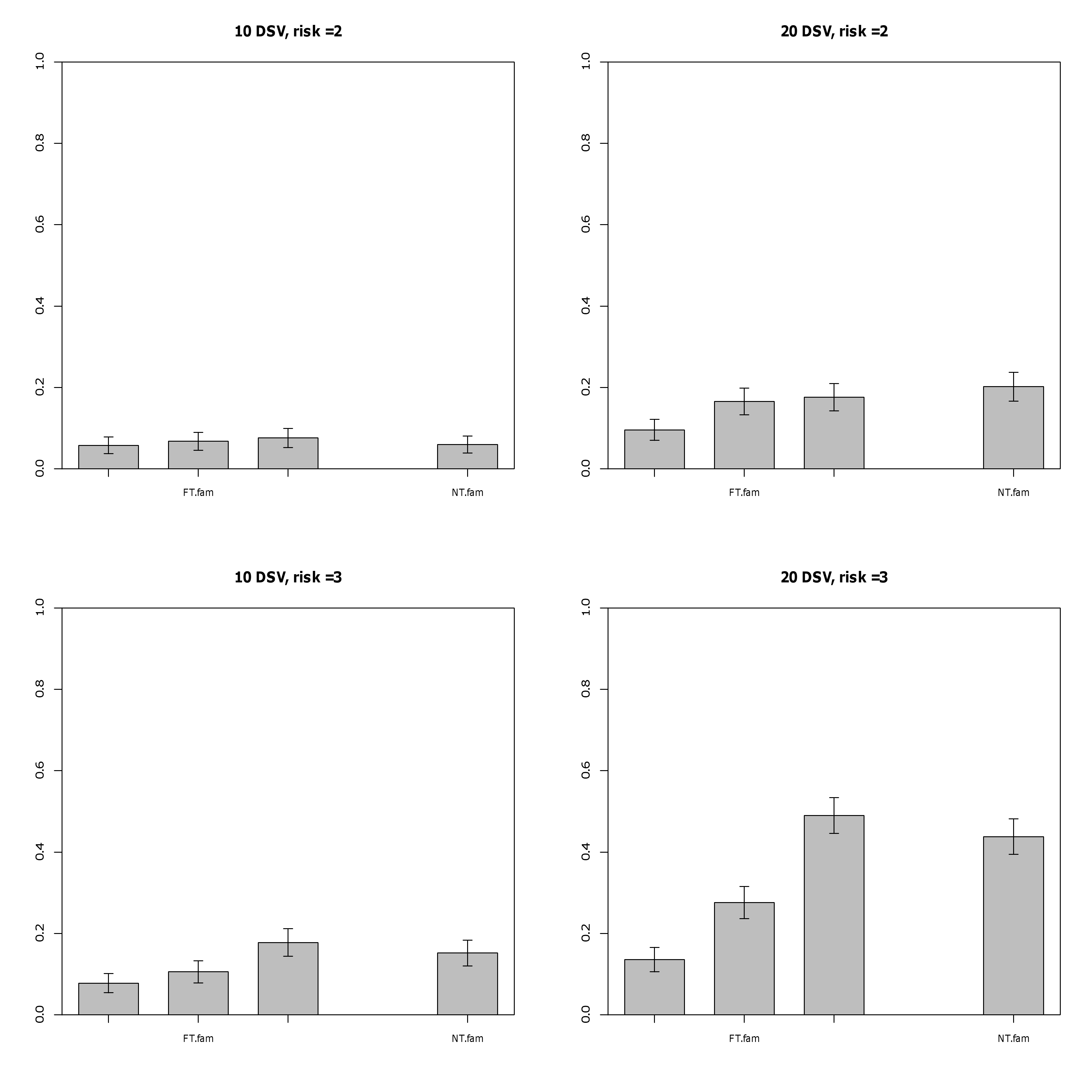

Supplement: Figure S4 — Power at 0.05 level for discordant sibpairs - No population stratification is present and . # of cases = 500. DSV’s have frequency less than 0.01 and equal effects. FT.fam - sibs with fixed threshold method using threshold 0.005, 0.01 and 0.05, NT.fam - sibs with weighted method using no threshold. (DOC) [file pone.0048495.s004.doc]
